# Inactivation of TCA cycle enhances *Staphylococcus aureus* persister cell formation in stationary phase

**DOI:** 10.1038/s41598-018-29123-0

**Published:** 2018-07-18

**Authors:** Ying Wang, Martin Saxtorph Bojer, Shilpa Elizabeth George, Zhihao Wang, Peter Ruhdal Jensen, Christiane Wolz, Hanne Ingmer

**Affiliations:** 1 Department of Veterinary and Animal Sciences, Faculty of Health and Medical Sciences, University of Copenhagen, Stigbøjlen 4, 1870 Frederiksberg C, Denmark; 2 Department of Medical Microbiology and Hygiene, Interfaculty Institute for Microbiology and Infection Medicine Tübingen (IMIT), University of Tübingen, Elfriede-Aulhorn-Straße 6, 72076 Tübingen, Germany; 3 National Food Institute, Technical University of Denmark, DK-2800 Kgs, Lyngby, Denmark

## Abstract

Persister cells constitute a small subpopulation of bacteria that display remarkably high antibiotic tolerance and for pathogens such as *Staphylococcus aureus* are suspected as culprits of chronic and recurrent infections. Persisters formed during exponential growth are characterized by low ATP levels but less is known of cells in stationary phase. By enrichment from a transposon mutant library in *S*. *aureus* we identified mutants that in this growth phase displayed enhanced persister cell formation. We found that inactivation of either *sucA* or *sucB*, encoding the subunits of the α-ketoglutarate dehydrogenase of the tricarboxylic acid cycle (TCA cycle), increased survival to lethal concentrations of ciprofloxacin by 10–100 fold as did inactivation of other TCA cycle genes or *atpA* encoding a subunit of the F_1_F_0_ ATPase. In *S*. *aureus*, TCA cycle activity and gene expression are de-repressed in stationary phase but single cells with low expression may be prone to form persisters. While ATP levels were not consistently affected in high persister mutants they commonly displayed reduced membrane potential, and persistence was enhanced by a protein motive force inhibitor. Our results show that persister cell formation in stationary phase does not correlate with ATP levels but is associated with low membrane potential.

## Introduction

Persister cells are phenotypic variants present in the bacterial populations that in the absence of heritable mutations or antibiotic resistance genes display remarkable antibiotic tolerance. They are detected as survivors when exposed to very high antibiotic concentrations often reaching 100 times the minimal inhibitory concentration (MIC) and they are observed in both exponential and stationary growth phases for multiple bacterial species and with several antibiotics^1,2^. During exponential growth, persister cells (designated type II) arise at very low frequencies whereas upon entry into stationary growth phase the fraction of persister cells (type I) increases substantially^3,4^. Commonly, persister cells are considered to be in a state of dormancy and several mechanisms have been proposed to explain the phenomenon. In *Escherichia coli* and *Salmonella*, dormancy was previously proposed to be stochastically induced by the action of the toxin-antitoxin (TA) systems that compromise key cellular functions and are activated by ppGpp or by the SOS response^5–10^. However, this model was recently disproven^11,12^. In fact, conditions that halt transcription, translation or ATP synthesis dramatically increase persister frequency from 0.01% to up to 10–100%^13^. Particularly, the cellular ATP level is important as depletion of ATP with arsenate increases the number of persister cells both in *E*. *coli* and in the human pathogen, *Staphylococcus aureus*^4,14^. In the latter organism, neither the stringent response nor TA loci contribute to persistence but rather persister cells express stationary phase marker genes, such as the capsular polysaccharide operon, suggesting that they are cells that during exponential growth have prematurely entered stationary phase with low ATP levels^4^.

Although the central role of bacterial metabolism in bacterial persistence has been documented by several reports, a uniform picture of the contribution has not emerged. In *E*. *coli*, fumarate accumulation and perturbations of the TCA cycle as well as the electron transport chain has been linked with increased persistence while others have found that inactivation of *sucB*, encoding α-ketoglutarate dehydrogenase and other genes of the TCA cycle reduced persister formation^15–17^. Tolerance to aminoglycosides is provided by low proton motive force (PMF) as persister cells are eradicated by conditions that enhance PMF and facilitates aminoglycoside uptake^18,19^. The varying contributions of metabolism to persister formation may in part be explained by different experimental setups but also by the significant differences in metabolism between organisms and growth conditions^13^. For example, in Gram-negative bacteria which are growing under laboratory conditions, such as *E*. *coli*, ATP is produced via oxidative phosphorylation driven by products generated in the TCA cycle while the low-passage counterpart produces ATP by substrate level phosphorylation; in the Gram-positive bacteria, *S*. *aureus*, the TCA cycle is repressed during exponential growth where glucose is metabolized to acetate with ATP generated by substrate level phosphorylation^20^.

*S*. *aureus* is known to give rise to a diverse range of serious diseases such as endocarditis, osteomyelitis and chronic and re-occurring infections that are difficult to treat with antibiotics. The clinical relevance of persister cells has been demonstrated by infection of a chronic wound model with *S*. *aureus* that could be eradicated with an acyldepsipeptide antibiotic, ADEP4 that killed persister cells^21,22^. With most studies of persisters having focused on the exponential growth phase we set out to determine if the already high level of persisters observed for *S*. *aureus* in stationary growth phase may be increased even further and if so which genes are contributing. For this purpose, we have taken a transposon library approach to identify genes that either by inactivation or overproduction increase persister cell frequency in stationary phase^23^. During enrichment, we found that inactivation of either *sucA* or *sucB* encoding subunits of α-ketoglutarate dehydrogenase dramatically increased persister cell formation as did inactivation of other enzymes of the TCA cycle or *atpA* gene. Commonly, all the mutants with enhanced persister levels displayed reduced membrane potential that contributes to PMF indicating that in stationary phase membrane potential becomes critical for persister cell formation or survival.

## Results

### Inactivation of α-ketoglutarate dehydrogenase increases persister cell formation in stationary phase

With the aim of identifying genes that contribute to persister cell formation in stationary phase in *S*. *aureus*, we constructed a high-density transposon (Tnp) insertion library in strain Newman employing the bacteriophage-based mariner transposon delivery system that allowed both gene inactivation and gene overexpression from an outward-facing promoter in the transposon element^23,24^. The coverage of Tnp insertions were assessed in 10 random library isolates and the results revealed that the 10 isolates carrying insertions at 10 independent sites were evenly distributed throughout the genome indicating random Tnp insertion (see Supplementary Table S1).

**Figure 1:**
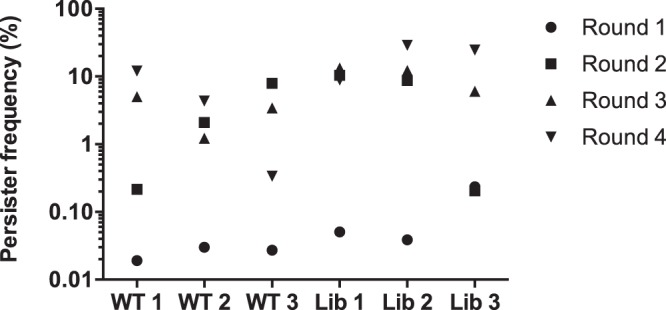
The enrichment of persisters with both *S*. *aureus* Newman wild type and transposon library. 24-hour culture of either wild type (WT 1–3) or Tnp library (Lib 1–3) was exposed to 100 × MIC of ciprofloxacin (MIC = 0.5 µg/ml) for another 24 hours before plating on TSA plates for CFU calculation. The same procedure was repeated consecutively for 4 times (Round 1–4) and the persister frequencies were recorded accordingly.

Library clones with increased frequency of persister cells formation in stationary phase were identified by an enrichment strategy where the Tnp library cells were repeatedly exposed to the fluoroquinolone drug, ciprofloxacin at a lethal dose of 100 times MIC^25^. The survivors were collected and inoculated into fresh medium and allowed to reach stationary phase before repeated exposure to ciprofloxacin. This treatment was repeated four times in total for pooled library clones as well as for the wild type strain Newman (Fig. 1) with the starting CFU/ml indicated in Supplementary Table S2. The persister frequency of strain Newman was approximately 1% by the end of enrichment with a few samples reaching 5–10% while the percentage of persisters from the Tnp library cultures exhibited more than 1000-fold increase, from the initial 0.01% to more than 20% after the four rounds of enrichment (Fig. 1). The increase in the fraction of persisters seen for wild type cells after enrichment implies that spontaneous mutations may enhance persister formation. Genome sequencing of one randomly picked clone confirmed this notion as it carried more than 200 mutations compared to the wild type cells prior to passage (data not shown).

**Figure 2:**
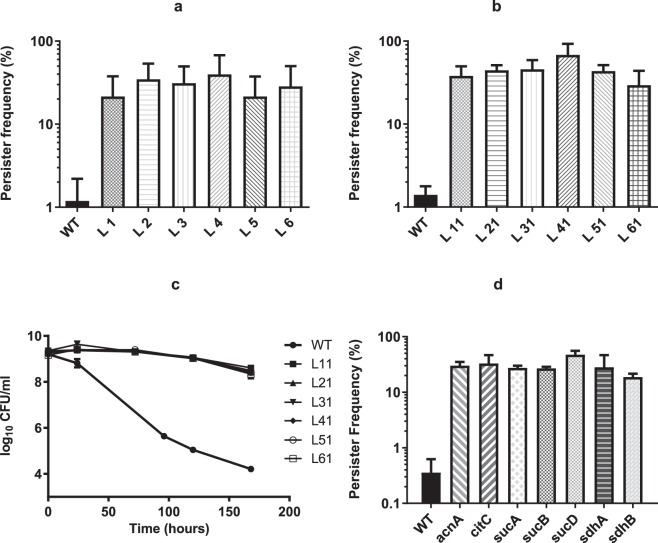
Persister frequency in the presence of ciprofloxacin or oxacillin. Persister frequency of 6 single colonies (L1-L6) of Tnp library (**a**) or their corresponding transductants (L11-L61) (**b**) in the presence of ciprofloxacin after persister enrichment was calculated by comparing the CFU calculation of 24-hour post and before the addition of 100 × MIC of ciprofloxacin (MIC = 0.5 µg/ml) (**c**) Survival of the transductants (L11-L61) challenged with 100 × MIC of oxacillin (MIC = 0.25 µg/ml) was recorded by CFU counting over 7 days. (**d**) Persister frequency of selected TCA cycle mutants (*acnA*, *citC*, *sucA*, *sucB*, *sucD*, *sdhA* and *sdhB*) when challenged with 100 × MIC of ciprofloxacin (MIC = 0.5 µg/ml). One wild type colony (WT) was included as control for all the experiments and starting CFU/ml are indicated in Supplementary Table S2. Three biological triplicates were included for each sample point and error bars represent standard deviation.

To determine if individual clones were enriched by repeated exposure of the Tnp library to high antibiotic concentrations, six colonies (named as L1-L6) were randomly picked by the end of the fourth round of enrichment to examine persister formation in individual clones. When treated with ciprofloxacin, all the six isolates exhibited persister frequencies between 20–60% compared to approximately 1% for the wild type (Fig. 2a). The transposon/chromosome junction was determined by sequencing and the result showed that in five of the six isolates (L2-L6) the transposon was inserted at one of three different locations within the *sucA* gene while in the last clone (L1) it was inserted in *sucB*. *sucA* and *sucB* encode subunits of α-ketoglutarate dehydrogenase, which is a key enzyme of the TCA cycle that catalyses the conversion of α-ketoglutarate to succinyl-CoA with the concomitant production of NADH and CO_2_ in an irreversible reaction^26^.

To rule out the possibility that secondary mutations within the library isolates contribute to the persister phenotype, the Tnp insertion mutations were transduced for all six clones to naïve wild type Newman cells to generate transductants, L11, L21-L61, carrying the individual mutations of the original library clones. All transductants yielded more than 30% persisters in comparison to 1% for the wild type when exposed to lethal concentrations of ciprofloxacin (Fig. 2b). To corroborate this finding and confirm that the enhanced persister cell formation is indeed due to inactivation of α-ketoglutarate dehydrogenase, an unmarked *sucA* deletion mutant (*ΔsucA*) was constructed. The *ΔsucA* mutant yielded more than 30% persisters when exposed to 100 times MIC of ciprofloxacin (see Supplementary Fig. S1). When the survival was followed for 48 hours after addition of ciprofloxacin, *ΔsucA* mutant exhibited improved survival compared to wild type which displayed the typical biphasic killing curve at the observed time points (see Supplementary Fig. S2). Additionally, the enhanced persister formation was also observed when the mutations were transduced into the *S*. *aureus* strains, SA564 and RN6607, which indicates that the current findings are not confined to strain Newman (see Supplementary Fig. S3). Further, we examined if the mutants influenced persister cell formation during exponential growth and found that in this growth phase the inactivation of *sucA* or *sucB* did not have any effect (data not shown) agreeing with the notion that in *S*. *aureus*, TCA cycle activity is low during exponential growth but de-repressed upon entry into stationary phase^20^.

Persister cells have been observed with other antibiotics than ciprofloxacin and therefore we examined if inactivation of α-ketoglutarate dehydrogenase increased survival during treatment with lethal doses of oxacillin (Fig. 2c). Because there was no discernible differences in persister generation between wild type and library isolate transductants after a 24-hour treatment, the effect of oxacillin on bacterial survival was monitored over a 7-day period. The counting of colony forming unit (CFU/ml) showed that there was less than 1 log CFU reduction of the library isolate transductants while wild type dropped from 10^9^ to 10^4^ CFU/ml after the 7-day treatment with 100 × MIC of oxacillin. Furthermore, the MICs of all library transductants towards ciprofloxacin and oxacillin were identical to that of wild type cells (see Supplementary Table S3), demonstrating that the increased persister cell formation is not a result of general changes in susceptibility to antibiotics. All together, these experiments show that inactivation of α-ketoglutarate dehydrogenase increases dramatically stationary phase persister cell formation in *S*. *aureus*.

### Inactivation of TCA cycle genes increases persister cell formation in stationary phase

α-ketoglutarate dehydrogenase is an enzyme of the TCA cycle and to address if TCA cycle activity in general influences persister formation, we examined persister frequency of mutants obtained from a sequence-defined transposon mutant library carrying insertional inactivations of *sucD*, *sdhA*, *sdhB*, *acnA and citC*^27^. Importantly, all of these mutants exhibited increased persister frequency (20–60%) paralleling that observed for the *sucA* and *sucB* mutants, when compared with wild type cells (<1%) in stationary growth phase (Fig. 2d). While it is unclear why our enrichment strategy only identified *sucA* and *sucB* mutants and not mutants lacking other TCA cycle enzymes, it may be related to the observation that after 24-hour of cultivation, the optical density at 600 nm (OD_600_) of *sucA* mutant cells was higher than observed for the other TCA cycle mutants (see Supplementary Fig. S4), indicating that this mutant may have a growth advantage. In summary, our results suggest that decreased TCA cycle activity increases *S*. *aureus* persister cell formation in stationary phase.

### Increased persister cell formation is not caused by slow growth or low pH

The appearance of persister cells has been associated with arrested bacterial growth^28^ but for the transductants carrying *sucA* or *sucB* inactivation we only observed slight differences in cell densities upon entry into stationary phase (see Supplementary Fig. S5). Similarly, long-term survival in stationary phase was not notably different between mutants and the wild type throughout a 7-day period (see Supplementary Fig. S5). In addition, the persister assay was repeated with bacterial cultures after 48-hour cultivation to ensure that all cells had entered stationary phase and again, the persister percentage of *sucA* mutant remained significantly higher than for wild type cells (see Supplementary Fig. S6), suggesting that any potential differences between strains in entry into stationary phase did not influence the results.

**Figure 3:**
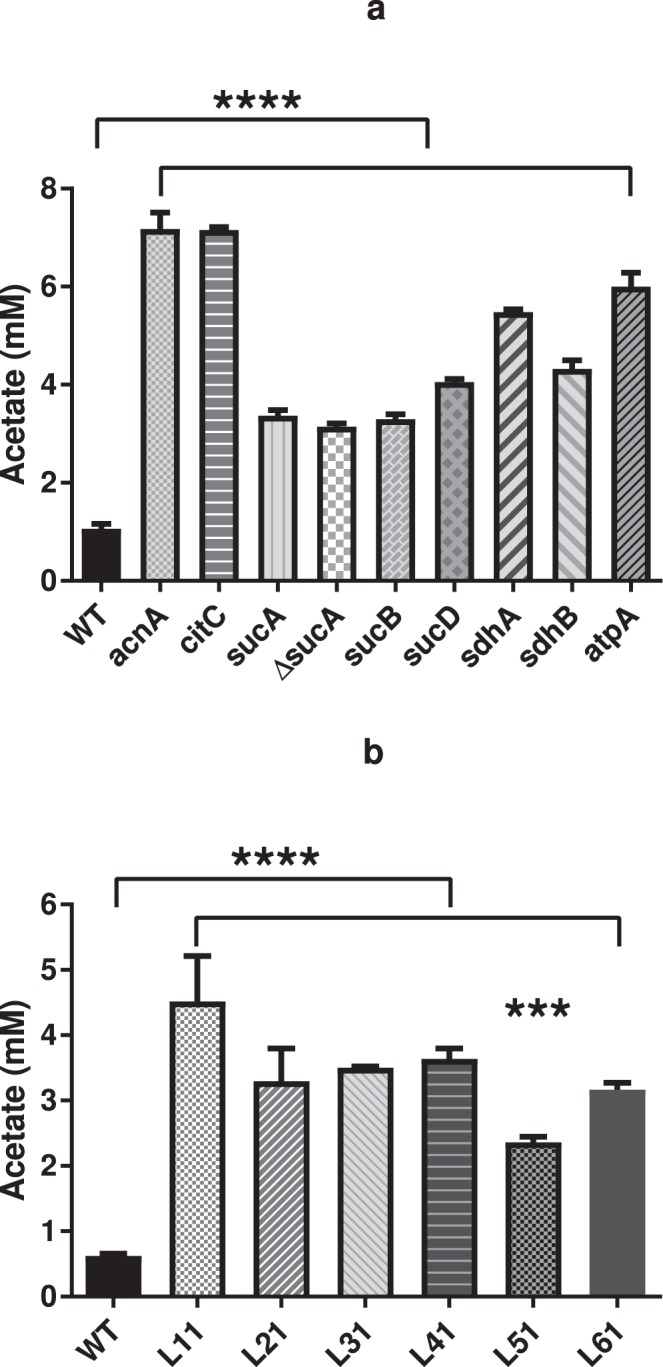
Acetate production by TCA cycle mutants (**a**) and transductants (L11-L61) (**b**). All strains were cultivated with TSB medium at 37 °C with shaking at 200 rpm and 1 ml of the culture was filtrated with 0.22 µm filter before the measurement with HPLC. The accumulation of acetate was calculated by comparing the concentration from early exponential phase (3-hour cultivation) and stationary phase (24-hour cultivation). There were 3 biological replicates for each sample and error bars represent standard deviation. The statistical comparison between wild type and mutants (**a**) or transductants (**b**) was determined with one way ANOVA followed by Dunnett’s test. The asterisks indicate significant difference at *P* < 0.05.

**Figure 4:**
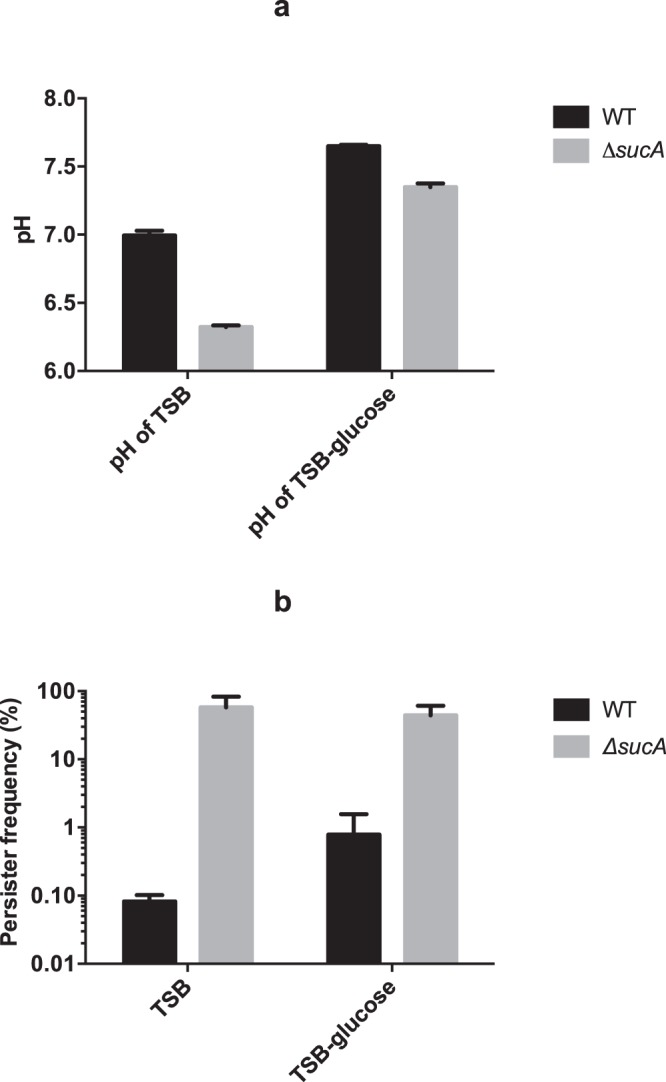
pH does not affect persister formation. (**a**) pH of cell-free supernatant. Wild type (WT) and *ΔsucA* were cultivated in TSB medium with or without glucose (TSB-glucose) for 24 hours and the pH of the supernatant was measured and compared; (**b**) the persister frequency of wild type (WT) and *ΔsucA* was calculated when cultivated with TSB medium or TSB medium without glucose (TSB-glucose). There were 3 biological replicates for each sample and error bars stand for standard deviation. Starting CFU/ml are indicated in Supplementary Table S2.

In contrast, we observed that culture supernatants of the TCA cycle mutants displayed lower pH (see Supplementary Table S4) and accumulated more acetate than wild type cells (Fig. 3a). This observation agrees with the fact that in *S*. *aureus*, the TCA cycle is required for post-exponential catabolism of acetate that has accumulated during exponential growth^20^. Similarly, the accumulation of acetate was also observed for all the transductants of library isolates (L11-L61) which have mutations of either *sucA* or *sucB* within TCA cycle (Fig. 3b). To examine if the enhanced persister cell formation was related to acidification of the growth medium, TSB medium without glucose was used to achieve comparable pH of supernatants from both *ΔsucA* and wild type cells in stationary phase (Fig. 4a). Importantly, in the absence of glucose where the pH of both mutant and wild type cultures was similar, the persister frequency of *ΔsucA* cells was still enhanced with 50% of the cells producing persisters while only 1% of wild type cells (Fig. 4b). These results show that low pH is not responsible for the increased persister cell formation in the TCA cycle mutants.

### Membrane potential is associated with persister cell formation

**Figure 5:**
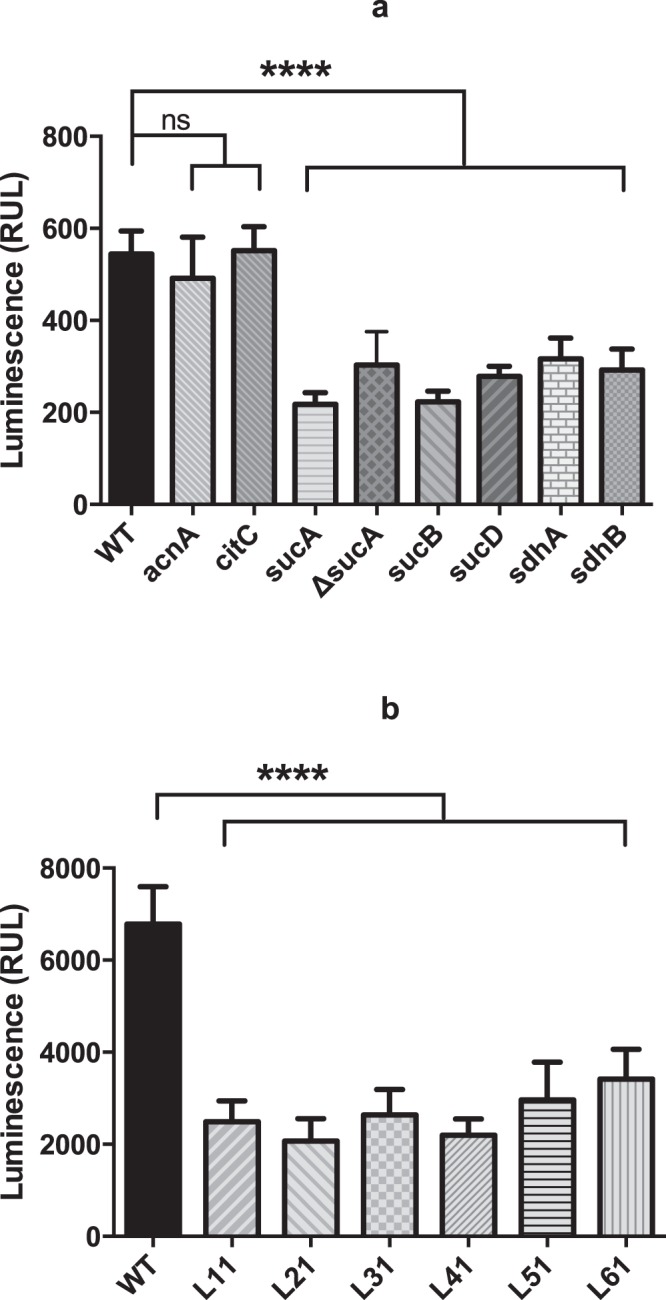
ATP is not the sole determinant of cellular persistence. (**a**) The ATP content of TCA cycle mutants (*acnA*, *citC*, *sucA*, *sucB*, *sucD*, *sdhA*, *sdhB* and *ΔsucA*) was measured after 24 hours of cultivation; (**b**) the ATP levels of wild type (WT) and transductants of Tnp library isolates (L11-L61) were measured after 24-hour cultivation. There were 3 biological replicates for each sample and 3 different reads of luminescence signal for each biological replicate. The statistical comparison between wild type and TCA cycle mutants (**a**) or Tnp isolate transductants (**b**) was determined with one way ANOVA followed by Dunnett’s test. The error bars stand for standard deviation and the asterisks indicate significant difference at *P* < 0.05.

Recently, it was demonstrated that *S*. *aureus* persisters generated during exponential growth are characterized by low ATP level^4^. However, when we assessed the ATP content we found that while the majority of the TCA cycle mutants (*sucA*, *sucB*, *sucD*, *sdhA* or *sdhB*, Fig. 5a) including the library isolate transductants (L11-L61, Fig. 5b) contained about 50% of the wild type ATP level, two mutants, namely *acnA* and *citC*, encoding aconitase and isocitrate dehydrogenase, respectively, had similar ATP levels as wild type cells (Fig. 5a) even though their ability to form persisters was remarkably higher than wild type cells (Fig. 2d). This phenomenon may be explained by the fact that some amino acids can feed into the TCA cycle at the point of α-ketoglutarate, and therefore, the inactivation of upstream enzymes, either aconitase (*acnA*) or isocitrate dehydrogenase (*citC*), may not affect the downstream metabolic activity of TCA cycle including ATP production^26^.

**Figure 6:**
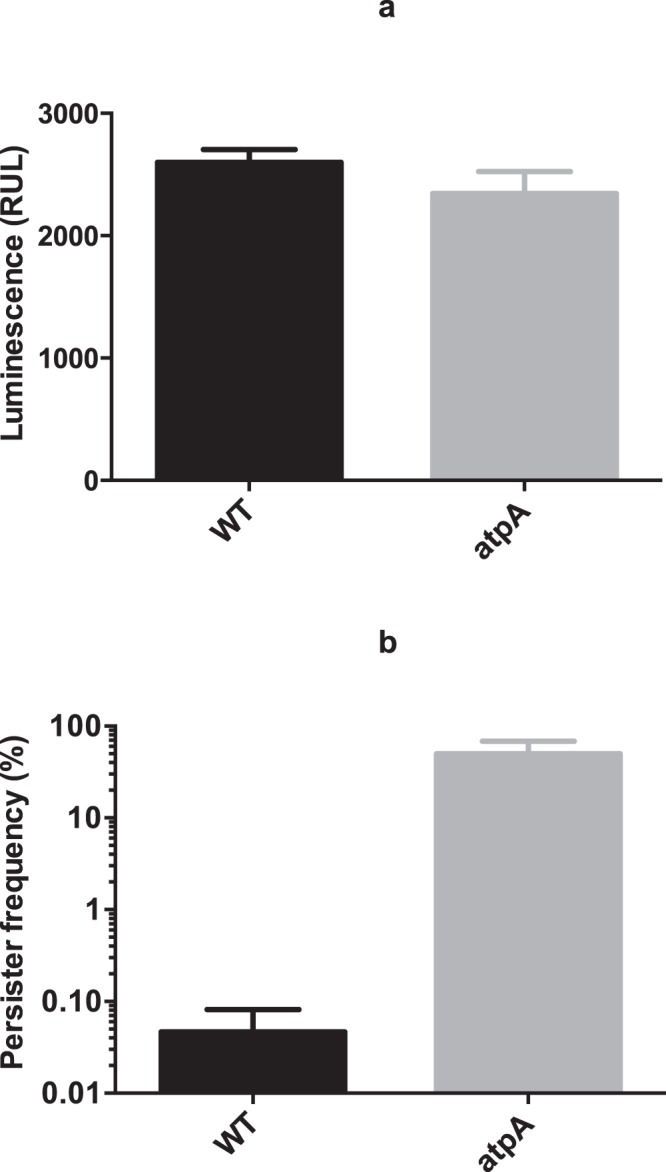
Comparison of ATP level (**a**) and persister frequency (**b**) between *S*. *aureus* Newman wild type (WT) and *atpA* mutant. For ATP measurement (**a**) 3 different reads of luminescence signal were taken for each biological replicate. Persister frequency (**b**) was tested with 100 × MIC of ciprofloxacin and the samples for ATP measurement were taken after 24 hours of incubation right before the addition of ciprofloxacin. There were 3 biological replicates for each sample and error bars indicate standard deviation. Starting CFU/ml are indicated in Supplementary Table S2.

During exponential growth ATP is generated by substrate level phosphorylation in *S*. *aureus*, but in stationary phase oxidative phosphorylation and the ATP synthase could contribute^20^. Therefore, we examined a mutant lacking *atpA* that encodes the α-subunit of F_1_F_0_ ATP synthase. As has been observed previously for other organisms we found that there was no significant reduction in ATP level of the *atpA* mutant when cultivated in rich medium (Fig. 6a) indicating that also in the post-exponential growth phase substrate level phosphorylation contributed to ATP production^29^. Strikingly, however, the inactivation of *atpA* increased persister formation approximately 1000-fold compared to wild type cells (Fig. 6b) underscoring the notion that ATP content is not decisive for *S*. *aureus* persister cell formation in stationary phase.

**Figure 7:**
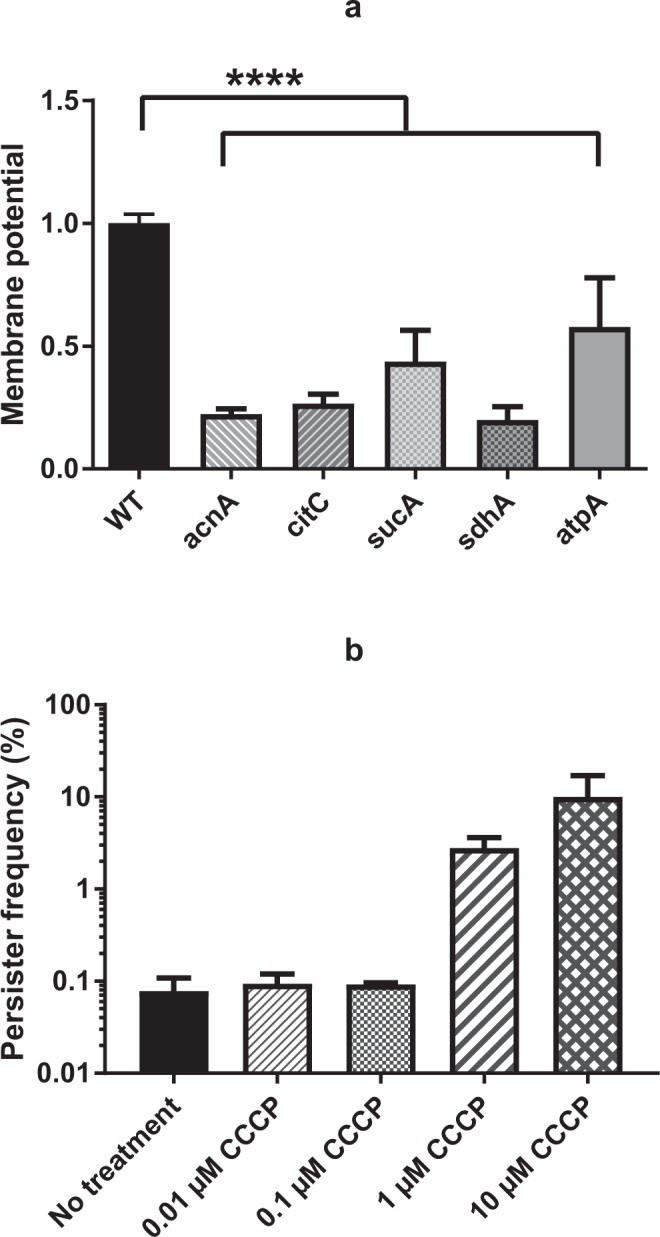
Measurement of membrane potential of selected mutants (**a**) and the persister formation in the presence of CCCP (**b**). (**a**) Membrane potential was measured for TCA cycle mutants (*acnA*, *citC*, *sucA* and *sdhA*) and *atpA* by flow cytometer after 30 minutes co-incubation with dye, DiOC_2_(3). (**b**) The persister frequency of *S*. *aureus* Newman wild type, challenged with 100 × MIC of ciproloxacin, was determined in the presence of CCCP, from 0.01 µM to 10 µM. There were biological triplicates for each sample and error bars represent standard deviation. Starting CFU/ml are indicated in Supplementary Table S2. The statistical comparison of membrane potential between wild type and selected TCA cycle mutants was determined with one way ANOVA followed by Dunnett’s test and the asterisks indicate significant difference at *P* < 0.05 (**a**).

Inactivation of the TCA cycle is likely to reduce the production of reducing equivalents that enters the electron transport chain and contributes to the PMF^30^. Therefore, we speculated that membrane potential, which contributes to PMF, may be related to persister cell formation. Indeed, when monitoring membrane potential using the fluorescent probe, DiOC_2_(3) and flow cytometry we consistently found that all TCA cycle mutants which exhibited increased persister cell frequencies displayed reduced membrane potential (Fig. 7a). Importantly, also the membrane potential of the *atpA* mutant was decreased compared to wild type cells (Fig. 7a). This result may indicate that wild type *S*. *aureus* cells in the post-exponential growth phase maintains PMF by extruding protons through the F_1_F_0_ ATP synthase with hydrolysis of ATP. The critical role of PMF for persister cell formation was lastly confirmed by addition of the PMF inhibitor, carbonyl cyanide m-chlorophenylhydrazone (CCCP) as expected it enhanced persister cell formation (Fig. 7b)^31–33^. Collectively, our results suggest that in stationary phase, the reduced proton motive force (PMF) characterizes *S*. *aureus* persister cells.

### *sucA* is expressed in stationary phase

**Figure 8:**
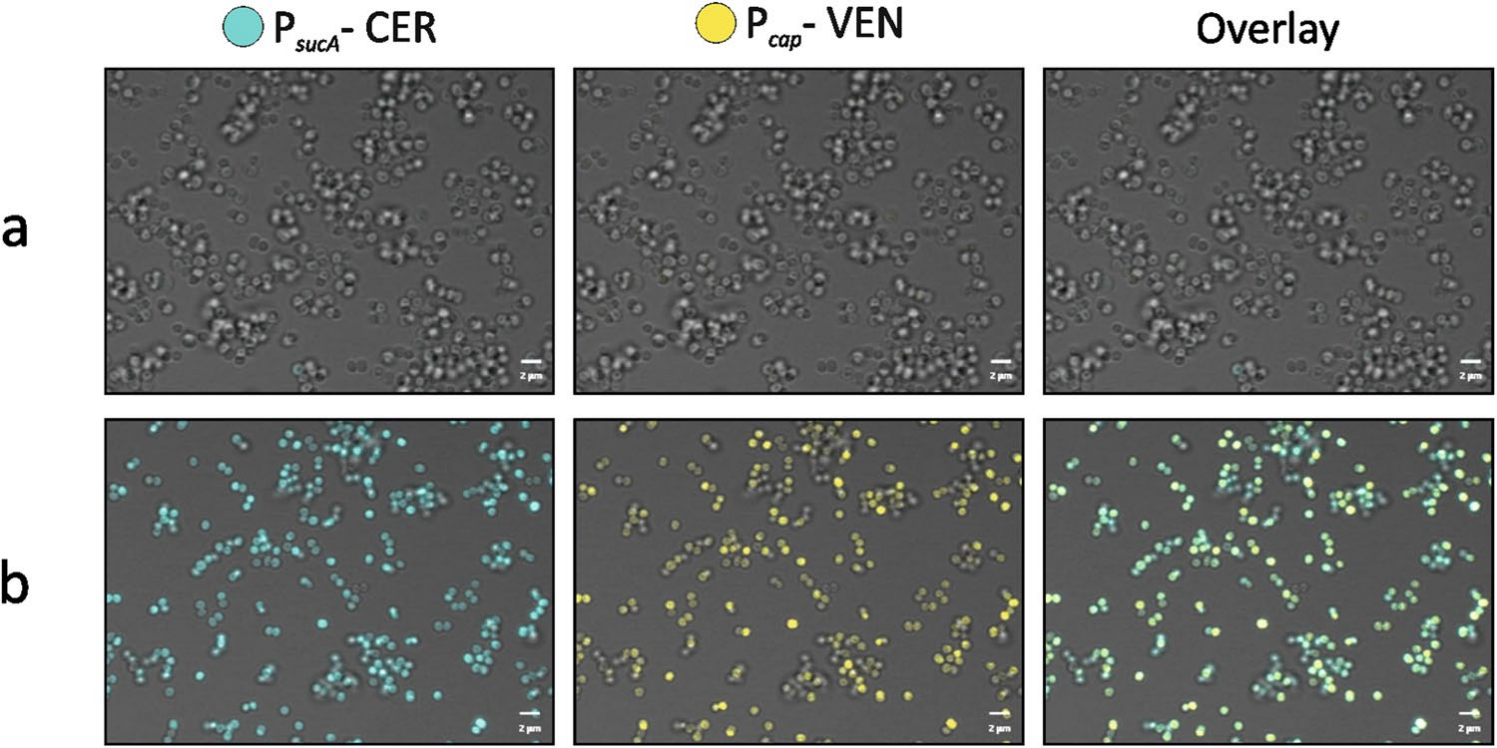
Expression of *sucA* in *S*. *aureus* Newman in exponential or stationary phase. The fluorescence expression of both P_*sucA*_ (CFP channel) and P_*cap*_ (YFP channel) in strain Newman was monitored at exponential (**a**) and stationary phase (**b**) respectively. The promoter activity of P_*sucA*_ appeared blue and P_*cap*_ activity appeared yellow.

In *S*. *aureus* TCA cycle activity is de-repressed in stationary phase^20^. To examine if this de-repression correlates with expression of TCA cycle genes, we fused the *sucA* promoter to the *cfp* reporter gene and monitored expression in exponential and stationary phase single cells (Fig. 8). While essentially no expression was observed in exponentially growing cells, *sucA* was clearly expressed in stationary phase cells. Similar expression pattern was observed for the *cap* promoter, an established stationary phase marker that is expressed along with TCA cycle activity^34^. In addition, we observed that expression of *sucA* varied between individual cells. These results show that TCA cycle genes are expressed in stationary phase and that variation in expression of *sucA* and possibly of other TCA cycle genes occurs. Thus, we speculate that in wild type cells, cell to cell variation in expression of TCA cycle genes in stationary phase determines persister cell formation.

## Discussion

When searching for mutants that in stationary phase gave increased persister frequencies we identified insertion mutants of *sucA* and *sucB*, which encode the subunits of the α-ketoglutarate dehydrogenase, a key enzyme of the TCA cycle that converts α-ketoglutarate to succinyl-CoA^35^. Importantly, also mutants lacking other genes of the TCA cycle as well as *atpA* encoding a subunit of the F_1_F_0_ ATP synthase displayed elevated persister cell frequencies. In contrast to our findings, Wang *et al*. reported that reduced activity of the electron transport chain and the TCA cycle decreased the number of persister cells when challenged with the fluoquinolone, levofloxacin^36^. To address the basis for this inconsistency, we examined our strains using their reported protocol. The result presented in Supplementary Fig. S7 shows that with the experimental approach by Wang *et al*., the *sdhA* (as well as *sucA*) mutant survived levofloxacin equally well as the wild type cells within a 5 days period. In contrast, when the persister assay was repeated with our standard protocol and using levofloxacin, both mutants (*sucA* and *sdhA*) exhibited higher persister frequencies compared to wild type cells (data not shown). Thus, we conclude that while the difference is not due to the antibiotics (ciprofloxacin vs. levofloxacin); there is a difference between the outcomes from the two protocols. We do, however, not see the same effect from an *sdhA* mutant in our strain background when using Wang’s persister assay protocol^36^, suggesting that strain choice is also important. Moreover, we have replicated our findings in different strain backgrounds (strains SA564 or RN6607, see Supplementary Fig. S3), supporting the generality of our findings. We also noted that, in Wang’s study^36^, the persister assay was carried out with static cultures in eppendorf tubes where the available oxygen is expected to be limited and rapidly depleted during cultivation. As an electron acceptor involved in electron transport chain, the scarcity of oxygen may restrict TCA cycle activity and oxidative phosphorylation, which could influence the outcome of their persister assay and potentially explain the differences compared to our results.

In the absence of antibiotics, a general link seems to exist between reduced TCA cycle activity and prolonged survival as inactivation of the aconitase resulted in extended stationary phase survival of *S*. *aureus*^37^. Also, small colony variants of *S*. *aureus* are resistant to aminoglycosides due to mutations in TCA cycle or electron transport chain genes and they display reduced membrane potential^38,39^. Recently, ATP content was reported to be a determining factor for whether or not exponentially growing cells become persisters^4,14^. However, in stationary phase cells of *S*. *aureus* we did not observe this correlation. In *S*. *aureus*, metabolism changes dramatically from exponential to stationary phase with the TCA cycle being inactive during exponential growth and ATP generated by substrate level phosphorylation whereas in stationary phase cells TCA cycle activity is de-repressed as acetate is being catabolized^20,40^. Consequently, persisters formed in exponential and stationary phase may be different and the genes contributing in the two phases may also vary. However, for all mutants with increased stationary phase persister cell frequencies in response to ciprofloxacin we observed a lower membrane potential than for wild type cells. Reduced membrane potential may for the TCA cycle mutants arise from reduced flow of electron donors to the electron transport chain while for the *atpA* mutant it indicates that in *S*. *aureus* the ATP synthase is normally working in reverse and contributes to PMF by pumping out protons fuelled by ATP hydrolysis. This may be necessary to get rid of the protons that at low pH enter the cell together with acetate which has accumulated during exponential growth. Membrane potential has previously been linked with persister cell formation but primarily in response to aminoglycosides. In *Pseudomonas aeruginosa*, PMF was increased by mannitol and it leads to increased killing of biofilm associated persister cells by tobramycin while in both *E*. *coli* and *S*. *aureus*, aminoglycoside uptake was stimulated by increased PMF leading to enhanced killing of persister cells^18,19^. Our data indicate that reduced PMF in stationary phase may enhance persister formation not only in response to aminoglycosides but also to fluoroquinolones. In our experiments, however, the high-persister phenotype with β-lactam treatment was observed over a 7 days period instead of within 24 hours as was the case for ciprofloxacin, which indicates a difference in persister mechanism between the two antibiotic classes. Since β-lactam killing relies on active cell wall synthesis, such a difference could result from increased cell turn-over in the wild type compared to the TCA cycle mutants. We speculate that mutations within TCA cycle contribute to the maintenance of *S*. *aureus* β-lactam persisters rather than their generation.

The exact mechanism by which the TCA cycle and PMF contribute to persister formation in *S*. *aureus* remains unclear. We found that expression of *sucA* is high and variable in stationary phase cells and correlates with the expression of *cap*, an established reporter of the stationary growth phase. Thus, we anticipate that in wild type populations, individual *S*. *aureus* cells with low expression of TCA cycle genes may be prone to form persisters. However, this is only in stationary phase, as in exponential phase where *sucA* is not expressed and TCA cycle is repressed, persister cells form at low frequency and TCA cycle inactivation does not influence this frequency. But how does reduced PMF lead to dormancy? In *Salmonella enterica*, the TacT toxin promotes persister formation by halting translation and its activity is modulated by the NAD^+^-dependent CobB sirtuin deacetylase suggesting that NAD^+^/NADH levels may influence persister formation^9,41^. However, for *S*. *aureus*, TA systems are not involved in persister cells formation indicating that alternative killing mechanisms should be sought^4^. Another possibility could be that the reduced membrane potential leads to a halt in cell wall synthesis as was recently shown in *Bacillus subtilis*^42^. Future analysis will be required to determine how reduced PMF leads to a persister state in *S*. *aureus*.

## Methods

### Strains, growth conditions and chemicals

A list of bacterial strains, plasmids and primers used in this study can be found in Supplementary Table S5. All the *S*. *aureus* strains, transposon library isolates and corresponding transductants, TCA cycle mutants and *atpA* mutant were all cultivated in Tryptic Soy Broth (TSB, Oxoid, Denmark) at 37 °C and 200 rpm of shaking in 12-ml centrifuge tube or plated on Tryptic Soy Agar (TSA, Oxoid, Denmark) at 37 °C of incubation unless otherwise indicated. All the plasmids and strains used for Tnp library construction were kindly provided by Timothy C. Meredith and supplemented glucose minimal medium (SGMM) was prepared as described previously^23^. TCA cycle mutants of *sucA*, *sucB*, *sucD*, *sdhA*, *sdhB*, *acnA* (NWMN-1263), *citC* and *atpA* were provided by bei Resources^27^. Ciprofloxacin, oxacillin, adenosine 5′-triphosphate disodium salt hydrate (ATP), carbonyl cyanidem-chlorophenylhydrazone (CCCP) and other mentioned chemicals were from Sigma-Aldrich (MO, USA). All restriction endonucleases and T4 ligase were from NEB BioLabs (MA, USA).

### Construction of transposon library and identification of Tnp insertion sites

Transposon library was constructed in *S*. *aureus* Newman as reported previously^23,24^: (1) Tnp donor lysates were prepared by infecting TM43-45 with Ø11-FRT and the Tnp-plasmid packaging efficacy was determined by tittering these phage lysates on both RN4220 and TM19; (2) recipient strains were obtained by transforming plasmids pOrf5 Tnp^+^ or pOrf5 Tnp^−^ into *S*. *aureus* Newman; (3) the Tnp donor lysates were gently mixed with recipient strains in SGMM and incubated at 22 °C overnight; (4) the cells were pelleted and incubated with TSB medium for 2 hours at 30 °C before plated on TSA plates containing erythromycin (5 µg/ml). Subsequently, the plates were left at room temperature until visible growth of individual Tnp insertion mutants could be observed.

The identification of the Tnp insertion site of library isolates was performed as reported by Bae *et al*. by PCR amplification of the transposon/chromosome junction using primers Martn-F and Martn-ermR and an annealing temperature of 63 °C. After purification, the PCR products were sequenced with primer Martn-F^43^.

### Monitoring of P_*cap*_ and P_*sucA*_ activity and image acquisition

Approximately 10^8^ bacterial cells were harvested and re-suspended in 1 ml of pre-chilled 1x phosphate buffered saline (PBS) containing 3.7% formaldehyde. After 15 minutes of gentle mixing at room temperature, 500 µl of the fixed bacterial suspension was transferred to each well of a 24-well cell culture plate (Greiner Bio-One, Germany) lined with 12 mm diameter round coverslips. After centrifugation at 600 *g* for 5 minutes, coverslips were mounted onto slides with 3 µl fluorescence mounting medium (DAKO, Denmark).

Image acquisition was performed in the confocal mode of an inverted Zeiss LSM 710 NLO microscope equipped with a spectral detector and employing Zeiss Plan-Apochromat 63x/1.40 oil DIC M27 objective (Zeiss, Germany). The following excitation wavelengths, laser sources and detection spectra were used for the promoter activity experiments, gpCerulean: 405 nm/diode laser/454–516 nm; gpVenus: 514 nm/argon laser/519–621 nm. Images were exported in the different channels or overlays as 16-bit tagged image files. The exported images were assembled with CorelDRAW X7 and adjusted for brightness and contrast.

### Enrichment of the mutants with enhanced persistence

1 µl of pooled wild type or Tnp library was inoculated into 2 ml TSB medium for incubation at 37 °C with shaking at 200 rpm in 12-ml centrifuge tube, after 24 hours, 1 ml of the culture was taken out to be treated by 100 × MIC of ciprofloxacin (MIC = 0.5 µg/ml) at 37 °C for another 24 hours in 12-ml centrifuge tube. The culture was washed and re-suspended with 1 ml FK buffer (9 g/l sodium chloride), and then certain dilutions were plated on TSA plates to be incubated at 37 °C for 24 hours. The plates with 100–200 colonies were eluted with FK buffer and 2 µl of the suspensions were inoculated in 2 ml fresh TSB medium which then undergone the same treatment of ciprofloxacin. This procedure was repeated for four times in a row to enrich persister cells.

### Persister assay

2 µl of overnight culture of library isolates, TCA cycle mutants or wild type was inoculated into 2 ml TSB medium and incubated at 37 °C with shaking at 200 rpm for 24 hours in 12-ml centrifuge tube, then 1 ml of the culture was challenged by 100 × MIC of ciprofloxacin for another 24 hours still at 37 °C in 12-ml centrifuge tube. The persister frequency was determined by calculating the ratio between the CFU/ml counting of 24-hour post and right before the addition of ciprofloxacin by plating and incubating known dilutions of the samples on TSA plates at 37 °C for 24 hours. Aliquots of *ΔsucA* mutant and parent strain cultures were taken at 1, 3, 5, 24 or 48 hours post the addition of ciprofloxacin and the dilutions were plated on TSA plate at 37 °C for 24 hours for CFU determination. The transductants of Tnp library isolates were also tested with 100 × MIC of oxacillin (MIC = 0.25 µg/ml) and the CFU/ml was determined over 7 days.

To evaluate the effect of medium acidification on persister generation, TSB medium with or without glucose (fisher scientific, MA, USA) was used for the cultivation and the persister frequencies were compared between the cultures with these two media. CCCP dissolved in DMSO was added into bacterial culture right before the treatment of ciprofloxacin to test the effect of PMF on persister generation.

### Quantification of cellular ATP level

The ATP level of 24-hour cultures was tested with BacTiter-Glo^TM^ Microbial Cell Viability Assay kit (Promega, WI, USA) according to manufacturer’s instruction. 100 µl suspension of bacterial culture (10-fold diluted with TSB medium) was carefully mixed with equal volume of BacTiter-Glo^TM^ Reagent and incubated at room temperature for 5 minutes before the luminescence was recorded with emission at 560 nm. The value of each sample was the average of three independent reads and there were 3 biological replicates for each individual sample.

### Measurement of membrane potential

Membrane potential was assessed by *Bac*Light^TM^ Bacterial Membrane Potential Kit (Life Technologies, CA, USA) where 24-hour cultures were re-suspended with 0.5 ml filtered PBS, mixed with 10 µl of fluorescent membrane potential indicator dye, DiOC_2_(3) and incubated at room temperature for 30 minutes. The fluorescent signals were recorded by BD Biosciences Accuri C6 flow cytometer (BD Biosciences, CA, USA) counting 50,000 cells with the threshold of 16,000 at medium flow rate. The ratio between channel F3 (red fluorescence) and F1 (green fluorescence) was calculated with CFlow® (BD Accuri, CA, USA) to indicate membrane potential.

### Quantification of fermentation products

The concentration of acetate in the supernatant of cultures from both early exponential (3-hour cultivation) and stationary phase (24-hour cultivation) was determined by Ultimate 3000 HPLC system (Dionex, Sunnyvale, USA) equipped with an Aminex HPX-87H column (Bio-Rad, Hercules, USA) and a Shodex RI-101 detector (Showa Denko KK, Tokyo, Japan). The column oven temperature was set as 60 °C and the mobile phase consisted of sulfuric acid (5 mM) with a flow rate of 0.5 ml/minute.

### Statistical analysis

Results were represented as the mean of biological triplicates with standard deviation besides for the ATP measurements there were 3 independent luminescent reads for each biological replicate. Statistical significance was analyzed using one way ANOVA followed by Dunnett test with P < 0.05 in GraphPad Prism 7 (GraphPad software Inc., CA, USA).

### Data Availability

The datasets generated during and/or analysed during the current study are available from the corresponding author on reasonable request.

## Electronic supplementary material

Supplementary information
