# Supplementary material for: Inactivation of TCA cycle enhances Staphylococcus aureus persister cell formation in stationary phase
Source: Sci Rep. 2018 Jul 18;8:10849. doi: 10.1038/s41598-018-29123-0 (PMC6052003; doi:10.1038/s41598-018-29123-0)
Supplement: Supplementary file 1 — Supplementary information [file 41598_2018_29123_MOESM1_ESM.docx]

**Supplementary Information for**

**Inactivation of TCA cycle enhances *Staphylococcus aureus* persister cell formation**

**in stationary phase**

**Ying Wang^1^, Martin Saxtorph Bojer^1^, Shilpa Elizabeth George^2^, Zhihao Wang^3^,**

**Peter Ruhdal Jensen^3^, Christiane Wolz^2^, Hanne Ingmer^1,^***

^1^Department of Veterinary and Animal Sciences, Faculty of Health and Medical Sciences, University of Copenhagen, Stigbøjlen 4, 1870 Frederiksberg, Denmark

^2^Department of Medical Microbiology and Hygiene, Interfaculty Institute of Microbiology and Infection Medicine Tübingen (IMIT), University of Tübingen, Elfriede-Aulhorn-Straße 6, 72076 Tübingen, Germany
^3^National Food Institute, Technical University of Denmark, DK-2800 Kgs. Lyngby, Denmark

*Correspondence: Hanne Ingmer, [*hi@sund.ku.dk*](mailto:hi@sund.ku.dk)

**Methods**

Construction of *ΔsucA*, TCA cycle mutants and transposon library transduction

Construction of an unmarked *sucA* deletion in *S. aureus* strain Newman was achieved by the use of the temperature-sensitive shuttle vector pBASE6^1^. 500-700 bp chromosomal regions surrounding the gene were amplified by PCR using primer pairs: Up-sucA_fw-BgIII / Up-sucA_rev-BG and Dw-sucA_fw / Dw-sucA_rev-SacI, respectively. These products were subsequently used as template in a spliced overlap extension PCR combining the forward and reverse primers from the previous reactions, thus generating a deletion fragment (from start to stop codon) that was cloned into pBASE6 via *Bgl*II/*Sac*I. The resulting plasmid was purified from *E. coli* IM08B and transformed directly into *S. aureus* strain Newman at 30°C followed by chromosomal integration by plating on TSA plates containing chloramphenicol (10 µg/ml) at 44°C overnight^2^. Plasmid cross-out was performed by passage at 30°C followed by plating on TSA plates containing anhydrotetracycline (500 ng/ml). Colonies were replica-plated to select for sensitivity towards chloramphenicol and successful allelic exchange was screened by PCR amplification using primers delta-sucA_ctrl_F and delta-sucA_ctrl_R positioned outside the chromosomal region used for homologous recombination.

The mutations of all the TCA cycle mutants (*sucA*, *sucB*, *sucD*, *sdhA*, *sdhB*, *acnA* and *citC*), Tnp library isolates and *atpA* mutant were transduced into strain Newman by the same approach^3^. The mutations were identified by the methods suggested by Nebraska Transposon Mutant Library with primer Buster^4^.

Construction of P*_cap_* - P*_sucA_* reporter strains

A dual promoter-reporter protein fusion cassette comprising P*_cap_*-VEN and P*_tagH_*-CER was synthesized and cloned into pCG246^5^. The resulting plasmid was digested with *BamH*I/*EcoR*I in order to exchange P*_tagH_* with P*_sucA_*. The *sucA* promoter was amplified from *S. aureus* strain Newman using the primer pairs PsucAgibfor and PsucAgibrev and cloned by Gibson assembly (NEB, MA, USA) into the linearized plasmid to generate pCG593. The plasmid was verified by restriction digestion and the insert was verified by PCR using the primer pairs: PsucAfor and PsucArev, PsucAfor and P-CG246rev. The plasmid pCG593 was transformed into RN4220 and then transduced into strain Newman.

Bacterial growth and stationary-phase survival

Bacterial growth was determined by inoculating 25 µl of overnight cultures into 25 ml of TSB medium in 250-ml Erlenmeyer flasks to be incubated at 37°C and OD_600_ was measured every hour for 12 hours in total. The stationary-phase survival was detected by plating the dilution of 10 µl bacterial cultures and counting CFU/ml every 24 hours for 7 days in total.

**Table S1.** The randomness of transposon insertions within *S. aureus* Newman genome.

| **Isolates** | **Insertion flanking sequence within whole genome** | **Isolates** | **Insertion flanking sequence within whole genome** |
| --- | --- | --- | --- |
| **1** | 2694461-2695013 | **6** | 2265821-2266063 |
| **2** | 282167-282877 | **7** | 139754-140527 |
| **3** | 2354292-2355503 | **8** | 1414194-1414940 |
| **4** | 778125-777905 | **9** | 944890-945156 |
| **5** | 911289-911568 | **10** | 2619384-2620429 |

**Table S2.** CFU/ml counting in scientific notation right before the addition of antibiotics during persister assays presented in current study.

| Figures | | Samples | |  | |  | | |  | | |  | |  | |
| --- | --- | --- | --- | --- | --- | --- | --- | --- | --- | --- | --- | --- | --- | --- | --- |
| Fig. 1 | |  | |  | |  | | |  | | |  | |  | |
|  | | **WT 1** | | **WT 2** | | **WT 3** | | | **Lib 1** | | | **Lib 2** | | **Lib 3** | |
| Round 1 | | 3,72E+09 | | 3,35E+09 | | 2,82E+09 | | | 3,07E+09 | | | 5,10E+09 | | 6,35E+09 | |
| Round 2 | | 3,04E+09 | | 4,92E+09 | | 8,42E+09 | | | 2,51E+09 | | | 3,58E+09 | | 4,06E+09 | |
| Round 3 | | 7,00E+09 | | 5,60E+09 | | 5,82E+09 | | | 3,93E+09 | | | 3,71E+09 | | 4,63E+09 | |
| Round 4 | | 4,59E+09 | | 5,65E+09 | | 5,41E+09 | | | 3,75E+09 | | | 2,74E+09 | | 2,43E+09 | |
| Fig. 2a | |  | |  | |  | | |  | | |  | |  | |
| WT | | **L1** | | **L2** | | **L3** | | | **L4** | | | **L5** | | **L6** | |
| 5,86E+09 | | 5,73E+09 | | 2,69E+09 | | 3,16E+09 | | | 3,24E+09 | | | 4,16E+09 | | 4,52E+09 | |
| Fig. 2b | |  | |  | |  | | |  | | |  | |  | |
| WT | | **L11** | | **L21** | | **L31** | | | **L41** | | | **L51** | | **L61** | |
| 3,74E+09 | | 4,16E+09 | | 2,82E+09 | | 3,64E+09 | | | 2,76E+09 | | | 3,51E+09 | | 5,17E+09 | |
| Fig. 2d | |  | |  | |  | | |  | | |  | |  | |
| WT | ***acnA*** | | ***citC*** | | ***sucA*** | | | ***sucB*** | | ***sucD*** | | | ***sdhA*** | | ***sdhB*** |
| 2,97E+09 | 2,59E+09 | | 3,27E+09 | | 3,33E+09 | | | 3,16E+09 | | 2,41E+09 | | | 3,15E+09 | | 3,76E+09 |
| Fig. 4b | |  | |  | | |  | | | |  |  | |  | |
| TSB WT | | **TSB *ΔsucA*** | | **TSB-glucose WT** | | | **TSB-glucose *ΔsucA*** | | | |  |  | |  | |
| 2.33E+09 | | 2.04E+09 | | 1.77E+09 | | | 8.53E+09 | | | |  |  | |  | |
| Fig. 6b | |  | |  | | |  | | | |  |  | |  | |
| WT | | ***atpA*** | |  | | |  | | | |  |  | |  | |
| 3,80E+09 | | 2,67E+09 | |  | | |  | | | |  |  | |  | |
| Fig. 7b | |  | |  | | |  | | | |  |  | |  | |
| WT 1 | | **WT 2** | | **WT 3** | | |  | | | |  |  | |  | |
| 7,30E+08 | | 6,70E+08 | | 1,20E+09 | | |  | | | |  |  | |  | |
| Fig. S2 | |  | |  | | |  | | | |  |  | |  | |
| WT | | ***ΔsucA*** | |  | | |  | | | |  |  | |  | |
| 2,53E+09 | | 2,46E+09 | |  | | |  | | | |  |  | |  | |
| Fig. S6 | |  | |  | | |  | | | |  |  | |  | |
| WT | | ***sucA*** | | ***sdhA*** | | |  | | | |  |  | |  | |
| 3,26E+09 | | 1,16E+09 | | 8,11E+08 | | |  | | | |  |  | |  | |

**Table S3.** The minimum inhibitory concentration (MIC) of ciprofloxacin and oxacillin of *S. aureus* Newman wild type, Tnp library isolates (L1-L6) and corresponding transductants (L11-L61).

| **Strains** | **MIC of Ciprofloxacin (µg/ml)** | **MIC of Oxacillin (µg/ml)** |
| --- | --- | --- |
| ***S. aureus* Newman wild type** | 0,5 | 0,25 |
| **Tnp library isolates (L1-L6)** | 0,5 | 0,25 |
| **Transductants of Tnp library isolates (L11-L61)** | 0,5 | 0,25 |

**Table S4.** pH value of culture supernatants of *S. aureus* Newman wild type (WT), TCA cycle mutants (*sucA*, *sucB*, *sucD*, *sdhA*, *sdhB*, *acnA* and *citC*), *atpA* and *ΔsucA*. All the samples were tested with biological triplicates (which were designated as 1, 2 and 3).

| **Strains** | **pH** | **Strains** | **pH** | **Strains** | **pH** | **Strains** | **pH** |
| --- | --- | --- | --- | --- | --- | --- | --- |
| **WT 1** | 6,89 | ***sucD* 1** | 5,94 | ***acnA* 1** | 5,63 | ***ΔsucA* 1** | 5,60 |
| **WT 2** | 6,92 | ***sucD* 2** | 5,99 | ***acnA* 2** | 5,70 | ***ΔsucA* 2** | 5,61 |
| **WT 3** | 6,83 | ***sucD* 3** | 5,94 | ***acnA* 3** | 5,67 | ***ΔsucA* 3** | 5,65 |
| ***sucA* 1** | 5,67 | ***sdhA* 1** | 5,60 | ***citC* 1** | 5,63 |  |  |
| ***sucA* 2** | 5,72 | ***sdhA* 2** | 5,56 | ***citC* 2** | 5,63 |  |  |
| ***sucA* 3** | 5,72 | ***sdhA* 3** | 5,52 | ***citC* 3** | 5,65 |  |  |
| ***sucB* 1** | 5,65 | ***sdhB* 1** | 5,54 | ***atpA* 1** | 6,29 |  |  |
| ***sucB* 2** | 5,71 | ***sdhB* 2** | 5,62 | ***atpA* 2** | 6,31 |  |  |
| ***sucB* 3** | 5,66 | ***sdhB* 3** | 5,62 | ***atpA* 3** | 6,39 |  |  |

**Table S5.** Bacterial strains and primers used in this study.

| **Strains** | **Description/Genotype** | **Source** |
| --- | --- | --- |
| ***E. coli*** | | |
| IM08B | mcrA Δ(mrr-hsdRMS-mcrBC) φ80lacZΔM15 ΔlacX74 recA1 araD139 Δ(ara-leu)7697 galU galK rpsL endA1 nupG Δdcm ΩPhelp-hsdMS (CC8-2) ΩPN25-hsdS (CC8-1) | In house |
| ***S. aureus*** | | |
| Newman (wild type) | NTCT8178 | In house |
| RN4220  SA564  RN6607 | Derivative of NTCT8325, r^-^m^+^, partial *agr* defect  human *S. aureus* isolate  *agr* II prototype laboratory strain | In house  In house  In house |
| *sucA* | NE547 (SAUSA300_1306) | 4 |
| *sucB* | NE1391 (SAUSA300_1305) | 4 |
| *sucD* | NE1770 (SAUSA300_1139) | 4 |
| *sdhA* | NE626 (SAUSA300_1047) | 4 |
| *sdhB* | NE808 (SAUSA300_1048) | 4 |
| *acnA* (NWMN-1263) | NE861 (SAUSA300_1246) | 4 |
| *citC* (Icd) | NE491 (SAUSA300_1640) | 4 |
| *atpA* | NE1660 (SAUSA300_2494) | 4 |
| TM19 | RN4220 Δ*attB*Ø11::Orf5; *geh*::pTM304 Tet^R^ | 6 |
| TM43 | TM 17 (pTM401) Tet^R^ Erm^R^; Tnp P_cap_ donor strain | 6 |
| TM44 | TM 17 (pTM402) Tet^R^ Erm^R^; Tnp P_pen_ donor strain | 6 |
| TM45 | TM 17 (pTM403) Tet^R^ Erm^R^; Tnp P_tuf_ donor strain | 6 |
| **Plasmids** | **Description/Genotype** | **Source** |
| pORF5 Tnp^+^ | pTM378 with the cl-like repressor ORF5 from Ø11 | 6 |
| pORF5 Tnp^-^ | pTM381 with the cl-like repressor ORF5 from Ø11 | 6 |
| pBASE6 | tetracycline inducible suicide mutagenesis vector | 1 |
| pCG246 | *cat bla*, *E. coli*/*Staphylococcus* shuttle vector, pCN47 derivative | 5 |
| **Primers** | **Sequences** | **Source** |
| Martn-F | 5’-TTTATGGTACCATTTCATTTTCCTGCTTTTTC-3’ | 7 |
| Martn-ermR | 5’-AAACTGATTTTTAGTAAACAGTTGACGATATTC-3’ | 7 |
| Up-sucA_fw-BgIII | 5'-GATACAAGATCTCAATCAAATATGATGTGAAGAATAAG-3' | This study |
| Up-sucA_rev-BG | 5'-TTTACACCCTCCACAAAAATG-3' | This study |
| Dw-sucA_fw | 5'-CATTTTTGTGGAGGGTGTAAAGGGGAAATAAGTCATGCCAG-3' | This study |
| Dw-sucA_rev-SacI | 5'-GATACAGAGCTCCTCTAATAATTTTTTGGCAGCTG-3' | This study |
| delta-sucA_ctrl_F | 5’-CAATTTGATACGGATCTGACATC-3’ | This study |
| delta-sucA_ctrl_R | 5’-CATAACATTTGTCATGTCAACTTCG-3’ | This study |
| Buster | 5’-GCTTTTTCTAAATGTTTTTTAAGTAAATCAAGTAC-3’ | 4 |
| PsucAgibfor | 5’- TATCAAGCAAAGTGACAGGGATCCAAAAGTGAAATTAACAAAGGA -3’ | This study |
| PsucAgibrev | 5’- CATAAATAATCATCCTCCTAAGGAATTCAAAAATGTTGAAACGCT -3’ | This study |
| PsucAfor | 5’-AGTGAAATTAACAAAGGAACAACGT-3’ | This study |
| PsucArev | 5’- AAAAATGTTGAAACGCTTACAATGT-3’ | This study |
| P-CG246rev | 5’- TCAAGTCCAACCAACTCGCT-3’ | This study |

**
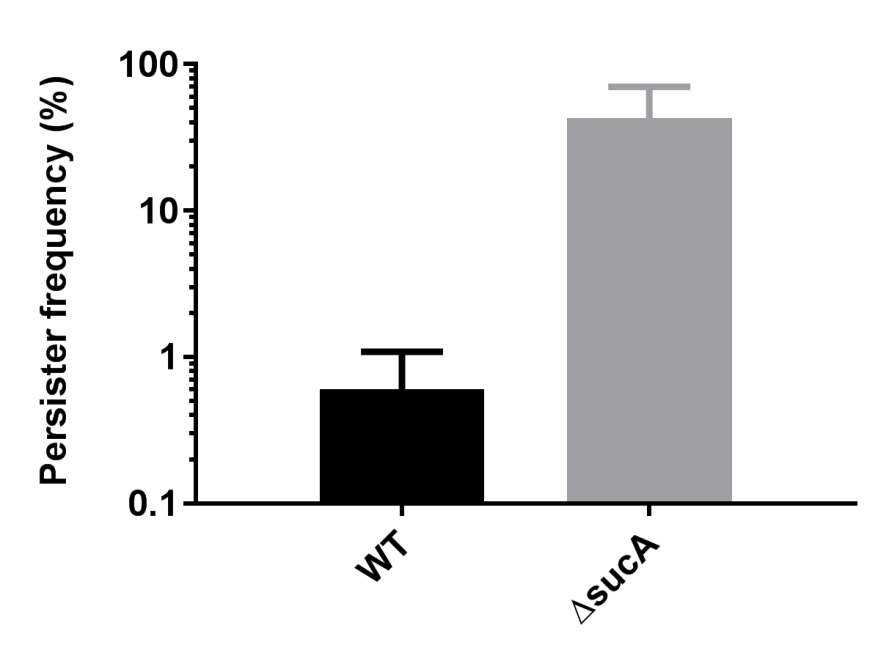
**

**Figure S1. Persister frequency compared between *S. aureus* Newman wild type (WT) and deletion mutant *ΔsucA* in the presence of ciprofloxacin.** The persister frequency was calculated by comparing the CFU calculation of 24-hour post and before the addition of 100 x MIC of ciprofloxacin (MIC=0.5 µg/ml). There were biological triplicates for each sample and error bars represent standard deviation.


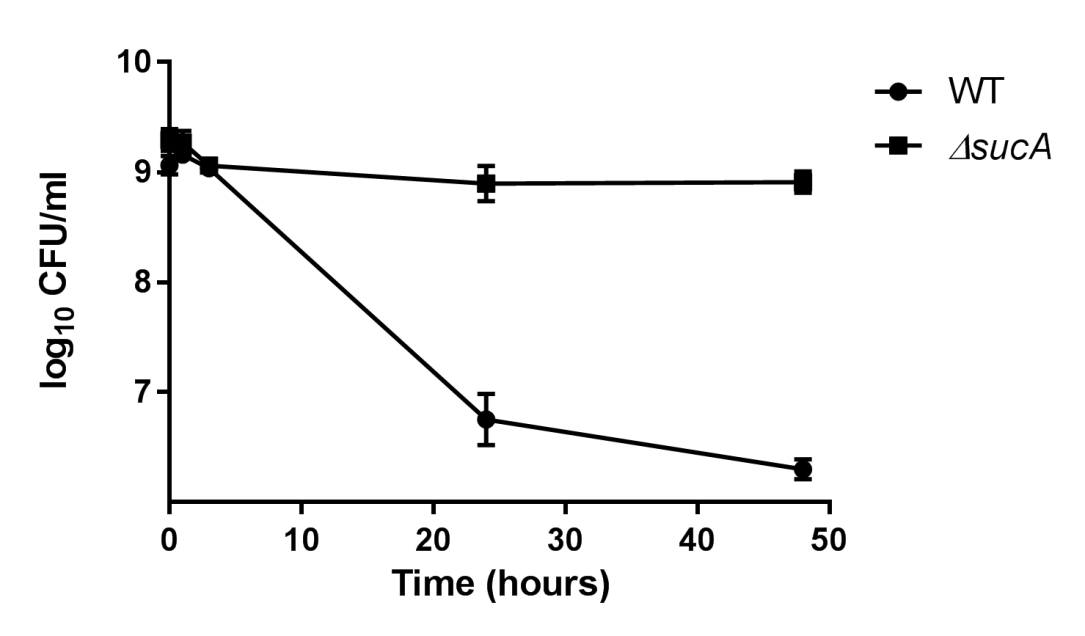


**Figure S2. The survival of *S. aureus* Newman wild type (WT) and deletion mutant** ***ΔsucA* mutant in the presence of ciprofloxacin.** The survival was determined by calculating CFU/ml of aliquots taken at 0, 1, 3, 24 and 48 hours post the addition of 100xMIC of ciprofloxacin (MIC=0.5 µg/ml). Biological triplicates for each strain were included and error bars indicate the standard deviation. Starting CFU/ml are indicated in Supplementary Table S2


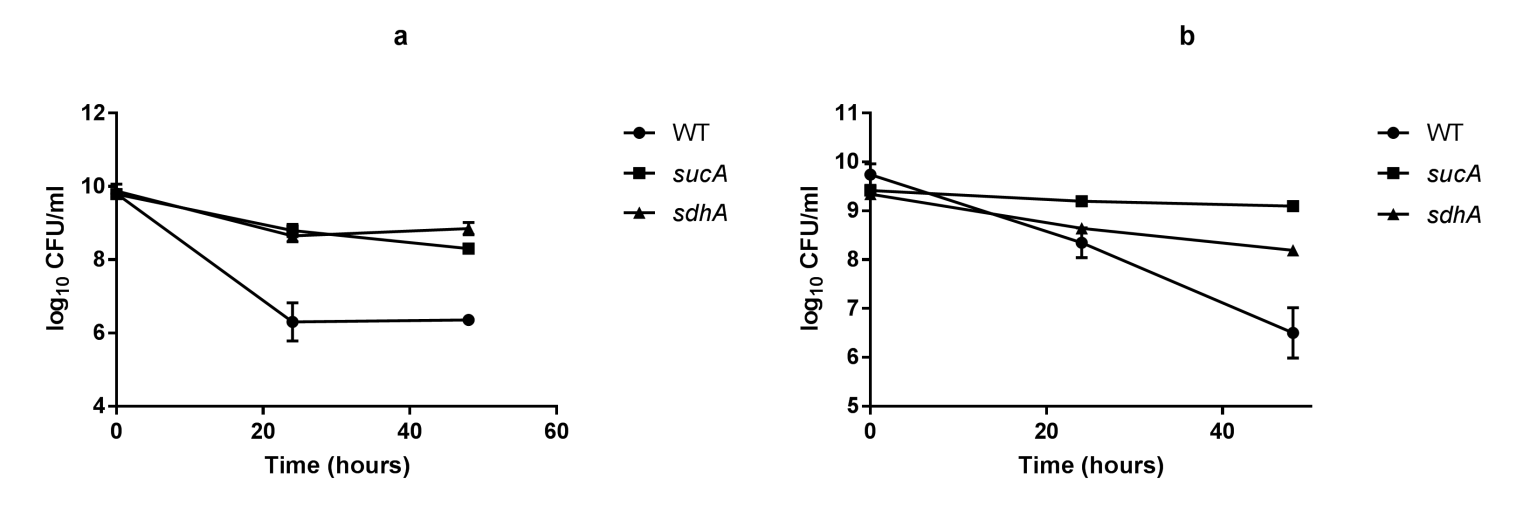


**Figure S3.** **Persister frequencies of *sucA* and *sdhA* mutants compared with wild type cells (WT) in strains SA564 (a) and RN6607 (b) in the presence of ciprofloxacin.** The persister assay was performed with both *sucA* and *sdhA* mutations transduced into strain SA564 or RN6607 comparing the CFU/ml right before and 24 hours post the addition of 100xMIC of ciprofloxacin. 3 biological replicates were included for each strain and the error bars indicate the standard deviation.


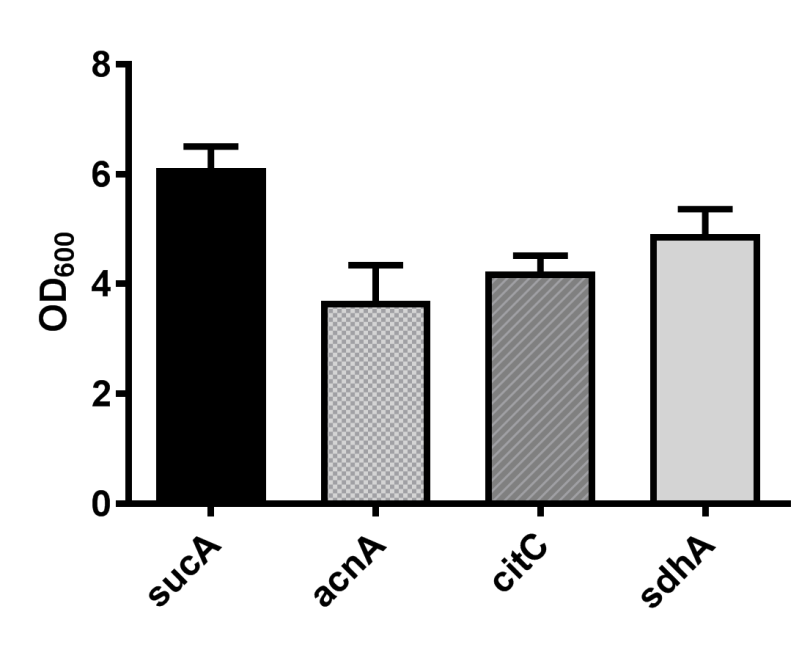


**Figure S4. Cell densities of selected TCA cycle mutants after 24-hour cultivation**. To compare the cell densities, optical density at 600 nm (OD_600_) was measured for *sucA*, *acnA*, *citC* and *sdhA* mutants after cultivated in TSB medium at 37°C and shaking of 200 rpm for 24 hours. There were 3 biological replicates for each sample and error bars represent standard deviation.


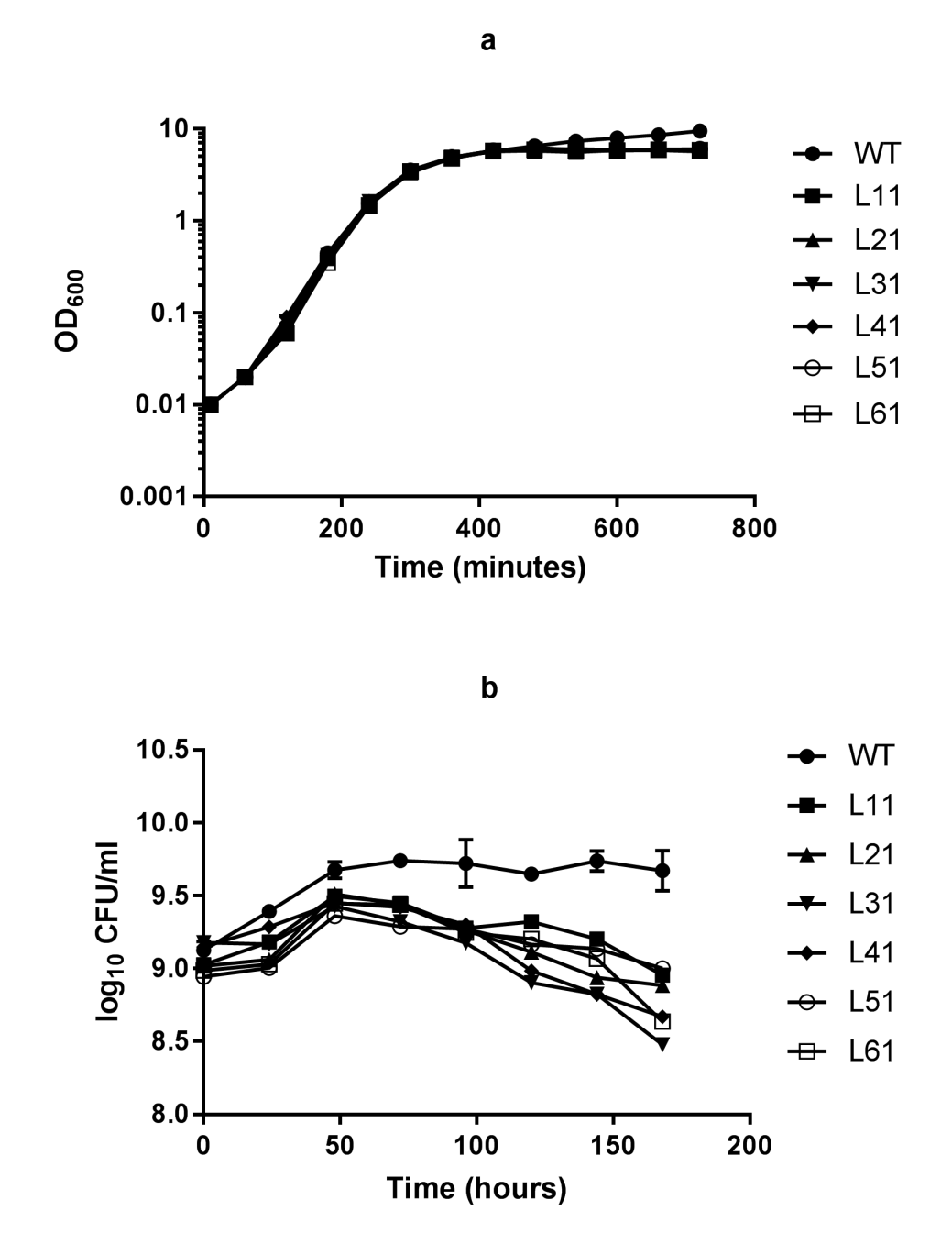


**Figure S5.** **Growth and stationary-phase survival of the transductants of Tnp library isolates (L11-L61) compared with wild type (WT).** The growth of L11-L61 in TSB medium at 37°C and shaking of 200 rpm was monitored over 12 hours (a) and the CFU/ml counting was examined every 24 hours for 7 days (b). The data shown were representative except the survival of WT (b) was from biological triplicates. Error bars stand for standard deviation.

**
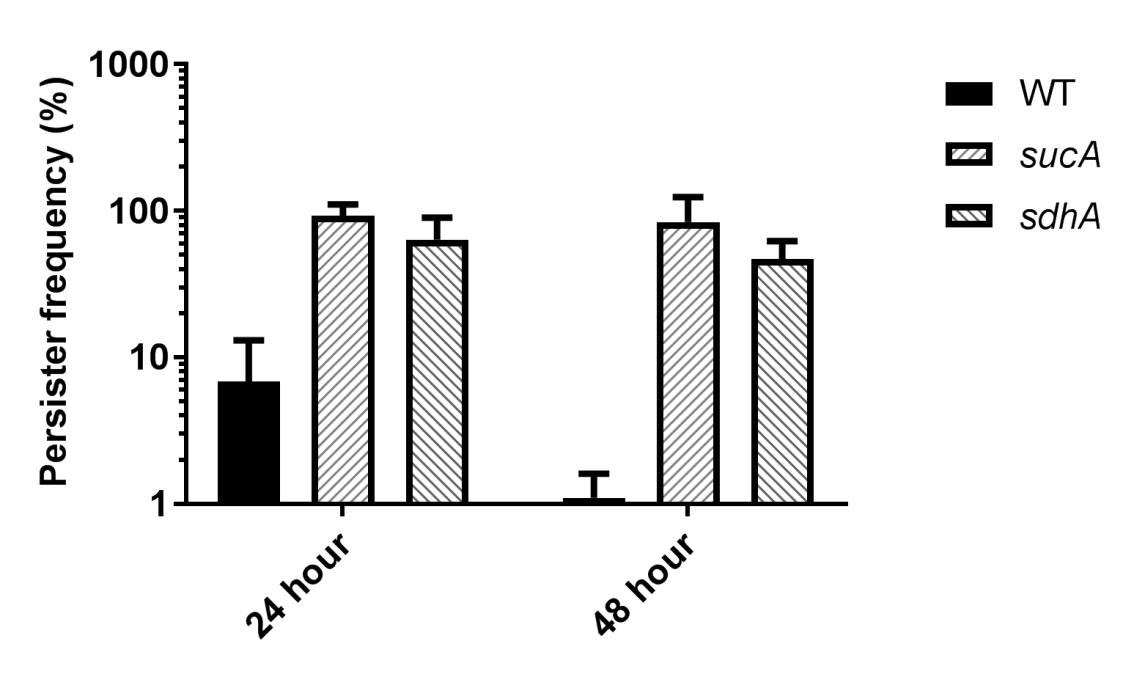
**

**Figure S6.** **Persister levels of 48-hour cultures challenged with ciprofloxacin.** *sucA*/*sdhA* mutants and wild type cells were cultivated for 48 hours before the addition of ciprofloxacin and the persister percentages of all samples were estimated by CFU/ml by plating on TSA plates right before and 24 hours or 48 hours after the addition of 100xMIC ciprofloxacin. Biological triplicates were included for each strain and error bars indicate the standard deviation. Starting CFU/ml are indicated in Supplementary Table S2

**
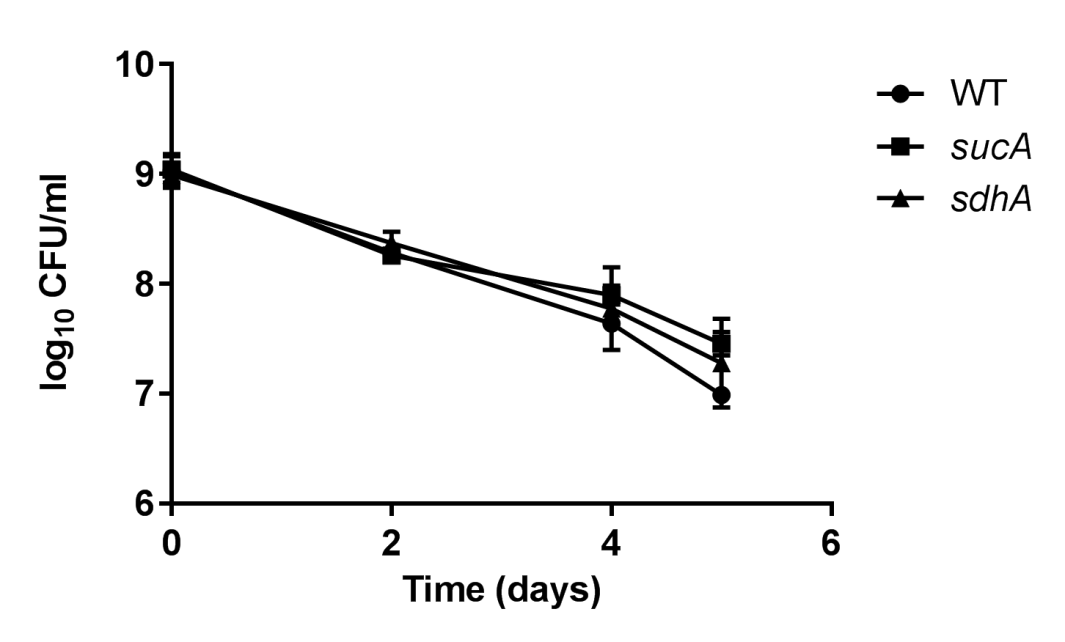
**

**Figure S7.** **Persister levels of *sucA* and *sdhA* mutants compared with wild type cells (WT) in the presence of levofloxacin using alternative protocol by Wang *et al*.^8^** The survival of *sucA* and *sdhA* mutants, as well as wild type cells, was monitored when exposed to levofloxacin (12.5 µg/ml) at designated time points with the protocol reported by *Wang et al*. Biological triplicates were included and error bars indicate standard deviation.

**References**

1 Geiger, T. *et al.* The stringent response of Staphylococcus aureus and its impact on survival after phagocytosis through the induction of intracellular PSMs expression. *PLoS Pathog* **8**, e1003016, doi:10.1371/journal.ppat.1003016 (2012).

2 Monk, I. R., Tree, J. J., Howden, B. P., Stinear, T. P. & Foster, T. J. Complete Bypass of Restriction Systems for Major Staphylococcus aureus Lineages. *MBio* **6**, e00308-00315, doi:10.1128/mBio.00308-15 (2015).

3 Lindsay, J. A. *Staphylococcus Molecular Genetics*. 112 (Caister Academic Press, 2008).

4 Fey, P. D. *et al.* A genetic resource for rapid and comprehensive phenotype screening of nonessential Staphylococcus aureus genes. *MBio* **4**, e00537-00512, doi:10.1128/mBio.00537-12 (2013).

5 Helle, L. *et al.* Vectors for improved Tet repressor-dependent gradual gene induction or silencing in Staphylococcus aureus. *Microbiology* **157**, 3314-3323, doi:10.1099/mic.0.052548-0 (2011).

6 Wang, H., Claveau, D., Vaillancourt, J. P., Roemer, T. & Meredith, T. C. High-frequency transposition for determining antibacterial mode of action. *Nat Chem Biol* **7**, 720-729, doi:10.1038/nchembio.643 (2011).

7 Bae, T. *et al.* Staphylococcus aureus virulence genes identified by bursa aurealis mutagenesis and nematode killing. *Proc Natl Acad Sci U S A* **101**, 12312-12317, doi:10.1073/pnas.0404728101 (2004).

8 Wang, W. *et al.* Transposon Mutagenesis Identifies Novel Genes Associated with Staphylococcus aureus Persister Formation. *Front Microbiol* **6**, 1437, doi:10.3389/fmicb.2015.01437 (2015).
